# 

**Published:** 1896-12-12

**Authors:** 


CADBURY'S COCOA
"The standard of highest purity at present attainable."—*Lancet.*

CADBURY'S COCOA

NO ALKALIES USED.

### THE HOSPITAL

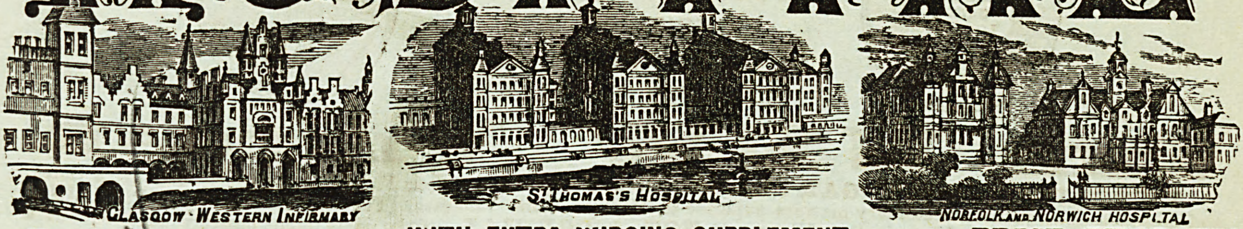

No. 533. Vol. XXI.

SATURDAY, DEC. 12TH, 1896.

WITH EXTRA NURSING SUPPLEMENT.
[Registered at the General Post Office as a Newspaper for transmission in the United Kingdom and Abroad.]

PRICE TWOPENCE.
Per Annum, 10s. 6d., post free.
Monthly Parts, 6d.; post free, 8d.
Ditto per Annum, 8s. 6d., post free.

